# Multi-scale Xception based depthwise separable convolution for single image super-resolution

**DOI:** 10.1371/journal.pone.0249278

**Published:** 2021-08-23

**Authors:** Wazir Muhammad, Supavadee Aramvith, Takao Onoye

**Affiliations:** 1 Department of Electrical Engineering, Faculty of Engineering, Chulalongkorn University, Bangkok, Thailand; 2 Multimedia Data Analytics and Processing Research Unit, Department of Electrical Engineering, Faculty of Engineering, Chulalongkorn University, Bangkok, Thailand; 3 Graduate School of Information Science and Technology, Osaka University, Suita, Osaka, Japan; University Tunku Abdul Rahman, MALAYSIA

## Abstract

The main target of Single image super-resolution is to recover high-quality or high-resolution image from degraded version of low-quality or low-resolution image. Recently, deep learning-based approaches have achieved significant performance in image super-resolution tasks. However, existing approaches related with image super-resolution fail to use the features information of low-resolution images as well as do not recover the hierarchical features for the final reconstruction purpose. In this research work, we have proposed a new architecture inspired by ResNet and Xception networks, which enable a significant drop in the number of network parameters and improve the processing speed to obtain the SR results. We are compared our proposed algorithm with existing state-of-the-art algorithms and confirmed the great ability to construct HR images with fine, rich, and sharp texture details as well as edges. The experimental results validate that our proposed approach has robust performance compared to other popular techniques related to accuracy, speed, and visual quality.

## 1 Introduction

Single image super-resolution (SISR) is more attractive in recovering the high-resolution (HR) output image from a degraded version of a low-resolution (LR) input image generating by a cheaper cost imaging framework within the limited environmental conditions. Recently, SISR, is a very interesting research space in the area of image and computer vision tasks, which is extensively applied in various applications such as; an object detection [1, 2], image segmentation [3, 4] and image classification [5, 6] purposes.

The better performance and higher accuracy of SISR have been encouraged in the area of an image, especially in medical imaging [7–9], face detection and recognition [10, 11], a high-definition television (HDTV) [12], video surveillance [13], satellite imaging [14] and autonomous driving technology [15, 16], where rich details information is greatly desired. Though, image SR is a highly challenging ill-posed inverse problem. Recently, a number of SISR approaches have been discussed to resolve the ill-posed inverse problem. These approaches can be subdivided into interpolation-based approaches (mostly employed as a pre-processing step to reconstruct the HR image), reconstruction-based approaches, and learning-based approaches. The interpolation-based approaches included as nearest neighbor-based interpolation [17], cubic interpolation [18] and edge guided interpolations. Although, above approaches are simple, and easy to implement, yet they suffer from accuracy shortcomings and are generating the jagged ringing artifacts. Reconstruction-based image super-resolution approaches [19–24] are mostly adopted previous information to narrow-down of the feasible solution which can get the benefit of reconstructing the fine details of edges and suppress the statistical noise effects [25]. However, these methods are time-consuming and rapidly degrading image reconstruction performance on 4× or 8× scale factor enlargements. Learning-based image SR methods are brought into focus by researchers due to outstanding performance and fast computation. Usually, such types of methods are using machine learning approaches to evaluate the relation between a low-resolution and a high-resolution input and output images during the training samples. Chang et al. [26] introduced the concept of neighbor embedding to take the benefit of similar patches generated locally for reconstructing the output of HR image from an input LR image patches. The researchers also used the idea of sparse signal recovery theory [27] and introduced the concept of sparse coding methods [14, 26–30] to solve the SISR problem. Meanwhile, reconstruction based approaches are combined with learning methods to reduce the jagged ringing artifacts and to improve the blurry results [28–31].

Currently, deep neural networks [32–39] provide significantly improved performance and led to dramatic changes in SISR. Furthermore, deep neural network approaches are very fast and accurate, but still, there are some limitations. However, existing deep convolutional neural network model stacked the convolution layer, side by side, to create the deeper network architecture, which leads to increase the computational cost and introduces the vanishing gradient problem during the training. Besides, a bicubic interpolation technique is used in existing deep convolutional neural network approaches as a step of pre-processing to upscale the low-resolution input image and incurs the new noises in the model. For the purpose of solving such issues and improving the quality of the LR image, we proposed a Multi-scale Xception Based Depthwise Separable Convolution for Single Image Super-resolution (MXDSIR) to generate the HR output image from the original LR input image.

In short, our key contributions are three folds across this paper:

- Inspired by the ResNet and Xception networks, we replaced regular convolution blocks with depthwise separable convolution blocks to achieve faster convergence during the period of training and to stop the vanishing gradient problem as well as easing the training complexity.
- The Rectified Linear Unit (ReLU) was replaced with the Parametric Rectified Linear Unit (PReLU) to activate the dead features, due to zero gradients.
- We introduced the new Xception block, which can detect the different image features information for rebuilding the HR image.

The remaining section is structured as follows. Section 2 presents a related work of image SR approaches. Section 3 and 4 explain our proposed method and its experimental results. Section 5 explained the conclusion.

## 2 Related work

The target of SISR image is to construct the visually pleasing HR output image. The first concrete deep learning-based approach for the SISR problem was suggested by Dong et al. [40] known as Super-Resolution Convolutional Neural Network (SRCNN) [40] and presented significant improvements over all previous SR methods. SRCNN [40] model used three convolution layers to predict the HR image. Wang et al. [41] introduced the sparse prior deep convolutional neural networks for image SR based approach, named as Sparse Coding Network (SCN) [41]. The performance of SCN [41] is better than SRCNN [40]. The major drawback of SCN [41] is the high computational complexity and also hinders its applications in real-time processing scenarios.

Dong et al. [42] proposed the improved and faster version of SRCNN [41] architecture to accelerate super-resolution image reconstruction, known as Fast Super-Resolution Convolutional Neural Network (FSRCNN) [42]. FSRCNN [42] has a modest network architecture, that depends on four CNN layers and one deconvolution layer for upsampling purposes and using the original input LR images without interpolation techniques. FSRCNN [42] has lower computational complexity and better performance as compared to SRCNN [41] but has a limited network capacity.

A very deep SR network (VDSR) [32] was proposed by Kim et al. [32] who was inspired by the Visual Geometry Group Network (VGG-net) implemented in the ImageNet for classification purpose [5]. VDSR [32] network reported the significant performance improvement over the SRCNN [41] network using the 20 CNN trainable layers. In order to ease the training complexity of a deeper model, they have used the global residual learning with a fast convergence rate. However, VDSR [32] network architecture does not use the actual pixel values but used the interpolated upscaled version of the image, which leads to more memory consumption and heavy computational cost. Kim et al. [33] proposed a Deeply Recursive Convolutional Network for image super-resolution (DRCN) [33] and uses the convolution layers multiple times. The key advantage of DRCN [33] is to fix the number of training parameters, although there are many number of recursions, the main deficiency is to slow the training process. The authors similarly used the skip connection with a recursive manner to optimize model performance. Mao et al. [43] extended the concept of residual type architecture and proposed Residual Encoder-Decoder Networks (RED) [43]. The RED [43] model used residual learning with symmetric convolution operation, which is trained on 30 layers and achieves the best performance. Therefore, such studies replicate the concept of “the Deeper the Better”.

Lai et al. [44] proposed a different network architecture for image SR is known as a deep Laplacian Pyramid Super-Resolution Network (LapSRN) [44], to generate the HR image. LapSRN [44] architecture depends on the different levels of the pyramid and each pyramid level is caused by a deconvolution layer as an upsample, but having the problem in scaling factor (fixed integer), which limits the flexibility of the model. Zhang et al. [45] suggested the denoising convolutional neural networks (DnCNNs), to accelerate the improvement of very deep neural network types architectures. DnCNN [45] follows the same architecture as SRCNN [40] and stacked the CNN with batch normalization (BN) layers followed by the ReLU activation function. Although the model provides favorable results, they are computationally expensive due to the use of the batch normalization layer. Zhao et al. [46] proposed a more flexible scaling factor to super-resolved the input LR image named as a gradual upsampling network (GUN) [46]. For Upsampling purposes the GUN [46] network architecture used the bicubic interpolation technique.

Tai et al. [47] introduced the idea of the deep recursive residual network (DRRN) [47] with 52 CNN layers. The authors introduced a stable training process for a deeper network with parallel architecture. Ledig et al. [34] employ a deep residual connection with 16 blocks using skip-connection to recover the upscaled version of the image. Lim et al. [48] proposed a method to develop deep SR architecture to increase the training efficiency of a model by eliminating the BN layers and their method to win the NTIRE2017 SR challenge [49]. Meanwhile, Tai et al. [50] suggested the deepest model, known as a persistent memory Network for image restoration purposes (MemNet) [50], in which multiple memory blocks are stacked to obtain persistent memory. Yamanaka et al. [51] presented a combined architecture of skip connection layers and parallelized CNN layers for development of a deep learning-based architecture for SISR and used mainly two networks, the first network is utilized for extracting the features of different levels and the second is the image reconstruction type network. This model is shallower than VDSR [32].

Han et al. [52] proposed the idea of Dual-State Recurrent Network (DSRN) [52], which exchanges the information from LR to the HR state. At each state, they update the signal information and then transmit to the HR state. Li et al. [53] used an adaptive feature detection process to obtain the features fusion at different scales, named as a multi-scale residual network [53]. This approach used the complete hierarchical type of feature information to reconstruct an accurate image super-resolution. Ahn et al. [54] proposed scale-specific upsampling type modules with multiple shortcut connections to learn residuals in LR feature space and to handle the multi-scale information with appropriate specific pathways. Zhang et al. [55] took a concatenated version of the low-resolution image with its degradation mapping type architecture named as super-resolution network for multiple degradations (SRMD) [55].

Wang et al. [56] introduced a dilated CNN network to enhance a receptive field without increasing the size of the kernel. The relative size of the receptive field increases in the case of shallow network type architecture. In dilated convolutional network for SR (DCNSR) [56] uses 12 layers to extract the contextual information efficiently. In [57], the authors proposed End-to-End Image SR via Deep and Shallow (EEDS) [57] CNN architecture and to replace the bicubic interpolation upsampling with the transposed upsampling layer. The HR image is obtained from deep as well as shallow branch simultaneously. Yang et al. [58] suggested a deep recurrent fusion network (DRFN) [58] for image super-resolution, which used the transposed convolution layer with large scale factors. Su et al. [59] proposed a novel type structure, that consists of several sub-networks for reconstructing the HR image progressively. In each sub-network, the input shall be utilized with the LR feature map and transposed convolution output will be fused with residuals to get the finer one. Wang et al. [60] solves the problem of single image SR using Heaviside Function with iterative refinement. The authors used the binary classification of images to reconstruct the HR image.

Hung et al. [61] proposed a super-sampling network (SSNet) [61] type architecture, which used depthwise separable convolution for image SR. In this architecture a number of parameters as well multiple operations can be significantly reduced by depthwise separable convolution technique. Barzegar et al. [62] introduced a small architecture to prevent the training problem in the deeper model. The design of a DetailNet architecture in such a way, that LR image information can be increased by any approach, then pass through main architecture to boost the perceptual quality of LR image. Hsu et al. [63] inspired by the capsule neural network to extract more potential features information for image SR. In this work authors designed two networks Capsule Image Restoration Neural Network and the Capsule Attention and Re-construction Neural Network (CARNN) [63] for image SR. The CARNN [63] network generates super-resolution features information efficiently. Liu et al. [64] proposed a new hierarchical convolutional neural network (HCNN) [64] architecture for SR purpose and to learn the features information at different stages. In this approach, the authors have used a three-step hierarchical process, which depends on the extraction of the edge branch, a branch of edge reinforcement, and the SR image reconstruction branch. Muhammad et al. [65] proposed multi-scale inception based super-resolution using a deep learning approach (MSISRD) [65] for image reconstruction. In this approach, the authors used the concept of asymmetric convolution operation to enhance the computational efficiency of the model and finally used the inception block to reconstruct the multiscale feature information for image SR.

Tian et al. [66] resolve the problem of instability during the training and proposed the new network architecture known as Coarse-to-fine CNN for SISR (CFSRCNN). The proposed network architecture consists of feature extraction, enhancement, construction and refinement of blocks to learn the robust image super-resolution model. The stacked feature extraction blocks are used to learn the short as well as long path features, and then finally fuses the learnable features by expending the effect of a shallow to deeper network to enhance the representing of the features.

Qiu et al. [67] proposed the method of multiple improved residual network (MIRN) image SR network architecture. In this network architecture deep residual network with different levels of skip connection is used to resolve the lack of correlation between the information of adjacent CNN layers. Stochastic gradient descent method (SGD) is used to train the MIRN network architecture. Lan et al. [68] proposed the new dense lightweight network architecture known as fast and lightweight network for SISR. This method addresses the problem of feature extraction and feature correlation learning.

The deep CNN based image SR network architectures used an excessive amount of CNN layers and parameters. Usually, used high computational cost and more memory consumption for training a SR model. To resolve these problems Tian et al. [69] proposed the lightweight enhanced super-resolution based SRCNN known as (LESRCNN). In this approach authors are used the three types of successive blocks as an information extraction, enhancement, and reconstruction block with information refinement block.

## 3 Proposed method

**Fig 1:**
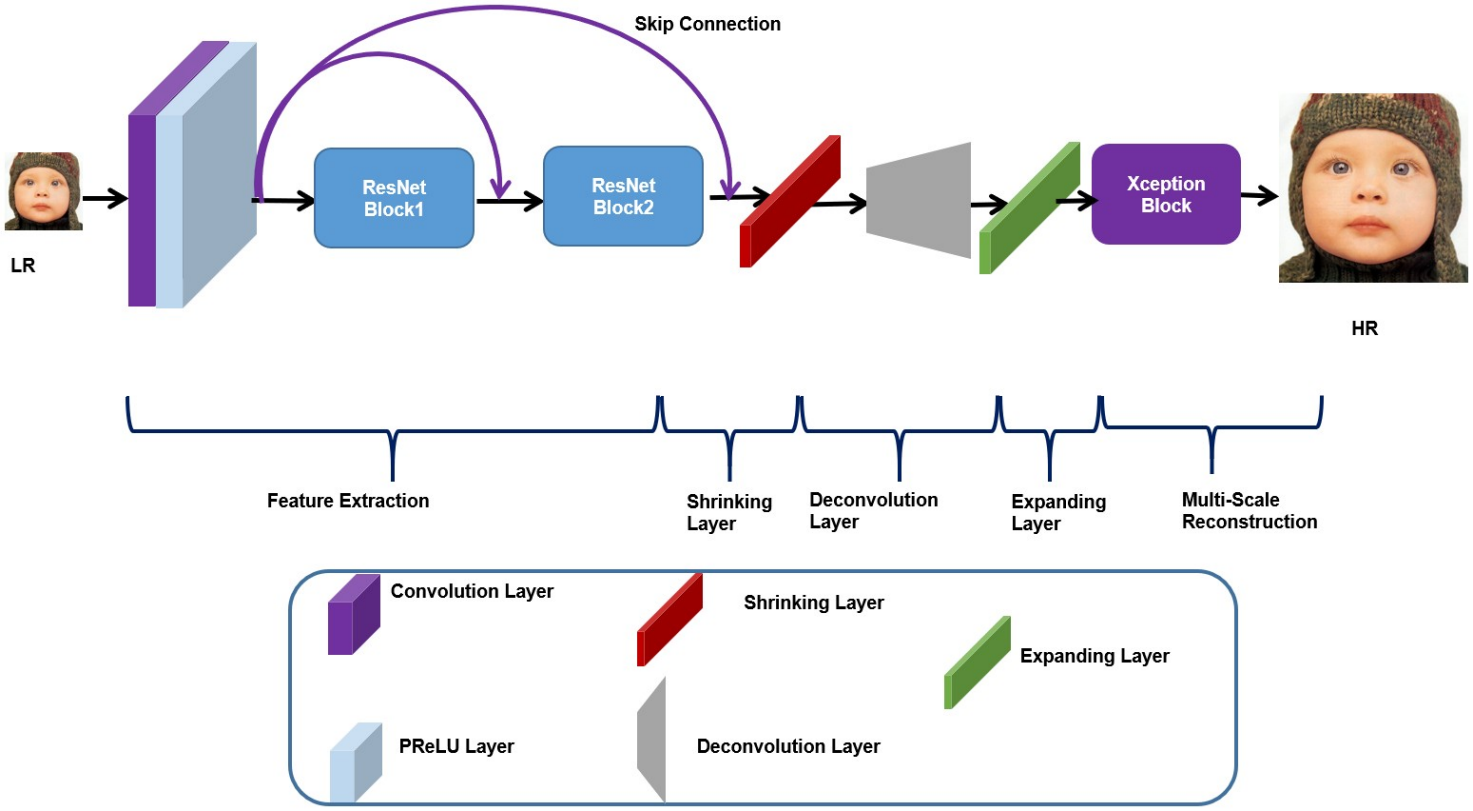
Proposed network architecture of Xception based single image super-resolution reconstruction.

In this section, we have discussed comprehensive details regarding our proposed network architecture for image SR based on ResNet and Xception blocks. Like the existing SISR methods, our proposed method is classified into five stages namely feature extraction, shrinking, upsampling, expanding, and multi-scale reconstruction, as shown in Fig 1.

### 3.1 Feature extraction

This part is similar to the previous methods but different from the input image. However, majority of the previous deep convolutional neural network type SISR approaches extract the features information from a bicubic interpolated upsampled version of the HR image. It is important to note that the bicubic interpolation technique damages vital information of LR image and introduces new noise in the model [57, 70]. In contrast, we have used an alternative strategy in our proposed model for extracting the features information directly from the LR image without using interpolation techniques.

Our initial feature extraction stage consists of one convolution layer and two ResNet Blocks with skip connection followed by Parametric Rectified Linear Unit (PReLU) [71] activation function. The said stage extracts the low, middle, and high-level features of information simultaneously. Inspired by VDSR [32], we have used one convolution layer of filter size 3 × 3 with 64 number of filters accompanied by the Parametric Rectified Linear Unit (PReLU) [71]. Mathematically, the convolution layer can be explained as:

$$\begin{array}{c} F_{l}\left(Y\right)=PReLU(W_{l}*F_{l-1}\left(Y\right)+B_{l}, \end{array}$$ (1)

where *F*_*l*_ denoted the resultant output features map, *B*_*l*_ denoted the biases of *l*^*th*^ layer.

$$\begin{array}{c} F_{l}=max\left(0,W_{l}*F_{l-1}+b_{l}\right), \end{array}$$ (2)

where *W*_*l*_ are the weights of the filter and *b*_*l*_ are the biases of the *l*^*t*^*h* layers, respectively. The output of the features map is denoted by *F*_*l*_ and “*” represents the convolution operation. The *W*_*l*_ supports *n*_*l*_ × *f*_*l*_ × *f*_*l*_ number of parameters, where, *f*_*l*_ indicates the filter size, *n*_*l*_ represents number of filters. The CNN layer and ResNet blocks have the same sizes of 3 × 3 × *c* of kernels which generate the “*c*” features map, where “*c*” represents 64 number of channels.

#### 3.1.1 PReLU

Earlier approaches used the convolution layers or blocks which were followed by the rectified Linear Unit (ReLU), like SRCNN [40] and VDSR [32]. These types of models have a fair response, but results are still not satisfactory, because, in most of the cases ReLU has a constant gradient. Whereas, in the proposed model, we have used the Parametric Rectified Linear Unit (PReLU) [71], which not only resolves the problem of constant gradient but also has a relatively faster speed of convergence during the training. Mathematically, PReLU [71] activation function can be explained as:

$$\begin{array}{c} PReLU\left(x_{i}\right)=max\left(x_{i},0\right)+a_{i}min\left(0,x_{i}\right), \end{array}$$ (3)

where *x*_*i*_ is the activation function of *i*^*th*^ layer input image, and the negative coefficient part of PReLU is denoted by *a*_*i*_, where *a*_*i*_ parameter is used as ReLU for zero value and PReLU for learnable purpose. The main purpose of PReLU is used to avoid the “dead features”, which is produced by zero gradients in the ReLU activation function. The resultant output feature maps using PReLU activation function can be written as:

$$\begin{array}{c} F_{l}\left(Y\right)=PReLU(W_{l}*F_{l-1}\left(Y\right)+B_{l}, \end{array}$$ (4)

where *F*_*l*_ denoted the resultant output features map, *B*_*l*_ denoted the biases of *l*^*th*^ layer.

#### 3.1.2 Feature extraction blocks

**Fig 2:**
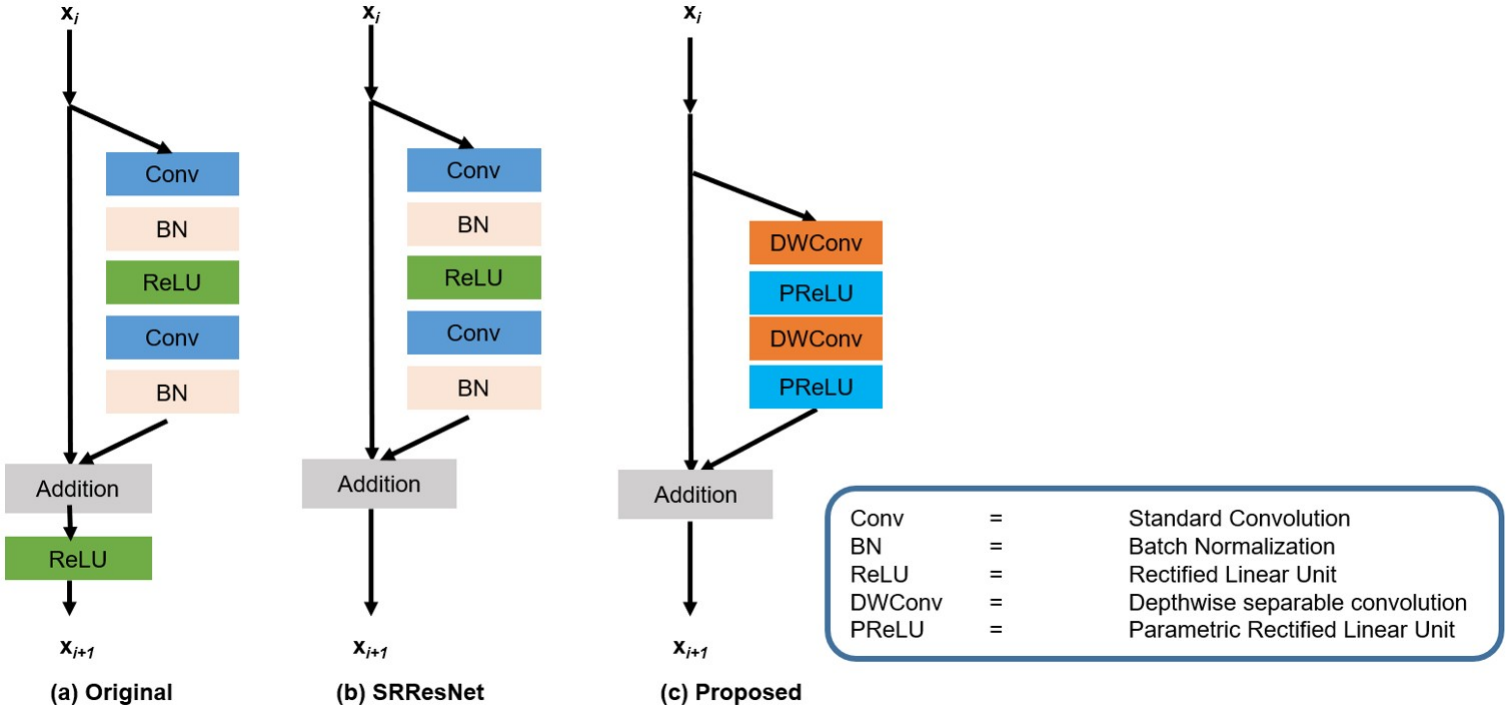
Comparison diagram of different ResNet blocks with the proposed ResNet block. (a) Original ResNet block. (b) SRResNet without final ReLU activation function. (c) Our Proposed ResNet block that removes the BN and replaces the regular convolution and ReLU activation function with depthwise separable convolution followed by the PReLU activation function.

The layer stacked, side by side, increases the network depth but reduces the transmission of information to the final layers [72]. Resultantly, the vanishing gradient problem arises in the model and the computational cost of the model is increased. He et al. [73] proposed the ResNet blocks to resolve the above-said problems. The ResNet blocks, these days, are extensively used in the deep learning type SISR image to reconstruct the HR image. Furthermore, the deeper ResNet architecture has a superior performance and is effectively used in the field of image SR [34, 48]. In our proposed method, we have used different residual skip connections which make fast training convergence and reduce the complexity of the model. In Fig 2; we have shown the comparison diagrams of the original residual skip [74] connection, SRResNet [34], and our proposed ResNet block.

The architecture of the ResNet block as expressed in Fig 2(a); uses a direct path and skip connection by way of transmitting the features information and the summed up resultant information followed by the ReLU activation function. SRResNet [34] block as indicated in Fig 2(b); uses the alternative strategy to remove the ReLU activation function and provides a simple and clear path from one block to another. Fig 2(c); shows our proposed ResNet block, which eliminates the Batch Normalization (BN) [74] layers for improving the efficiency of the Graphics Processing Unit (GPU) memory card and enhances the computational efficiency of the model. Furthermore, we replace the operation of regular convolution with depthwise separable convolution followed by point wise convolution and ReLU activation function with PReLU. The PReLU is used to avoid the problem of vanishing gradient and to reduce the training complexity as well as enhances the efficiency of the block. For the middle and the high-level feature extraction, we applied 2 ResNet blocks, each block consists of two 3 × 3 depthwise separable convolution kernels with 64 filters followed by PReLU nonlinearity.

### 3.2 Shrinking layer

If more features are directly applied to the transpose convolution layer, it will led to increase in computational cost as well as in size of the model. However, we have employed a one CNN layer as a shrinking layer before the deconvolution layer. This type of arrangement has also been observed in the latest convolutional neural network architectures for computer vision applications. Authors, proposed in [57, 65, 75] are using a shrinking layer for increasing the computational efficiency of the model.

### 3.3 Deconvolution layer

Researchers have suggested in [40, 57, 76] that the purpose of upscaling the LR image resolution before the initial layer is to increase the computational cost and damage critical information due to the fact that the processing speed is directly dependent on resolution of the image. Furthermore, the use of upscaled techniques before the initial layer does not provide additional information, however, introduces the jagged ringing artifacts in the SR image. We propose for generating the high-resolution image directly from the actual low-resolution feature domain to handle these types of problems. For this purpose, we have applied the deconvolution layer as an upscaling operation before the Xception block. The size of the deconvolution layer is 16 × 16 of stride that is equal to enlargement factors.

### 3.4 Expanding layer

The expanding layer performs the inverse operation of a shrinking layer and produces the HR image more accurately. Furthermore, if the HR image is directly reconstructed from LR features, the final reconstruction quality of the image will be poor. Therefore, after the deconvolution layer, we are applying the expanding layer to recover the original feature’s information smoothly.

### 3.5 Multi-scale reconstruction

#### 3.5.1 Depthwise separable convolution

Originally, depthwise separable convolution was proposed by Sifre [77] and was applied for image classification purposes. Factorizing a convolution operation is a form of depthwise separable convolution in which it converts regular convolution operation into a depthwise separable convolution operation followed by a pointwise convolution operation. The separable convolution operation performs a single filter per channel input and finally combines the linear input channels. The convolution process substitutes a factorized convolution layer with two layers; one is used for space filter, and the other is used for combining purposes. Thus, the depthwise separable convolution will sufficiently lessen both the number of parameters and size of the model. The regular type of convolution kernel takes three parameters such as; height (*h*), width (*w*), and input channel (*c*_*in*_) of an input feature map (*I*). The resultant convolution layer (*h* × *w* × *c*_*in*_) is applied as *K* × *K* × *c*_*in*_ × *c*_*out*_, where *c*_*out*_, is the number of output channels. The depthwise separable convolution depends on two convolution operations: depthwise separable convolution operation and pointwise convolution operation. Mathematically, the depthwise separable convolution operation can be written as:

$$\begin{array}{c} G\left(y,x,j\right)=\sum_{u=1}^{k}\sum_{v=1}^{k}K(u,v,j)\times I\left(y+u-1,x+v,j\right), \end{array}$$ (5)

where *K* represents the kernels of depthwise separable convolution operation of size *K* × *K* × *c*_*in*_. The *n*^*th*^ filter in the kernel *K* is applied on the *n*^*th*^ number of channels in the input feature map of *I* to reconstruct the *G* output feature map. While reconstructing new features, we apply the pointwise convolution. Mathematically, the pointwise convolution can be written as:

$$\begin{array}{c} O\left(y,x,l\right)=\sum_{j=1}^{c_{in}}G(x,y,j)\times P\left(j,l\right), \end{array}$$ (6)

where the size of the kernel of pointwise convolution operation is 1 × 1 × *c*_*in*_ × *c*_*out*_.

#### 3.5.2 Xception block

**Fig 3:**
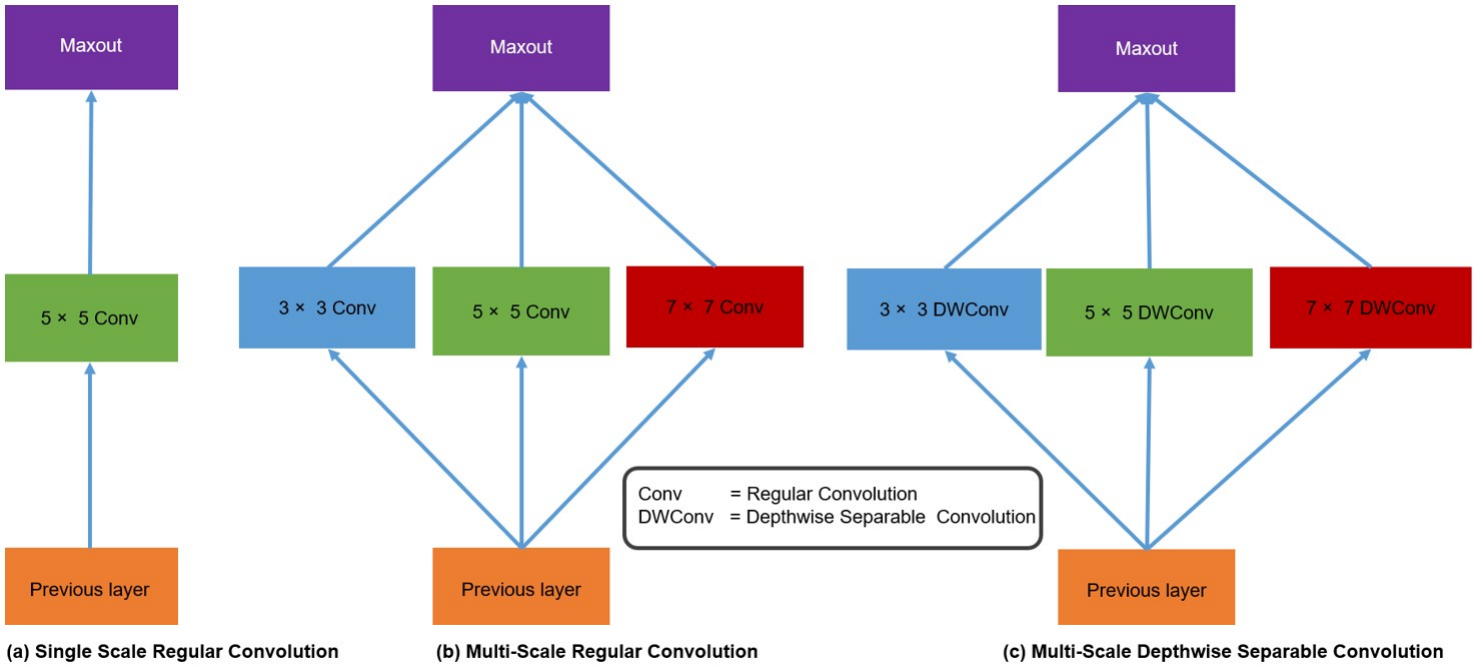
Comparison of a single scale, multi-scale regular and depthwise separable convolution blocks. (a) Single Scale Regular Convolution (b), Multi-Scale Regular Convolution, and (c) Multi-Scale Depthwise Separable Convolution (Our proposed).

In the final phase, we have employed a multi-scale Xception block that stands for a multi-scale Extreme version of Inception block, which is adopted from GoogLeNet [78] with a modified depthwise separable convolution better than Inception v-3 [79]. Multi-scale Xception block is used to choose the correct kernel size, as kernel size performs a pivotal role in model design, training procedure, and multi-scale reconstruction purposes. The larger size of the kernel is more suitable, when the features information is distributed globally, whereas the smaller size of the kernel is better, when features information is distributed locally. The Xception architecture employs this concept and includes more depthwise separable convolution on kernels of various sizes. Fig 3(a); shows a single scale regular convolution plain type of architectures, in which several convolution layers are stacked in a single straight-line path. Such type of architectures are implemented by a well-known image super-resolution methods, like SRCNN [40] and FSRCNN [42]. These types of architectures are easy in implementation, however, deeper network architecture has more memory consumption and enhances the network depth of the model. Fig 3(b); uses the regular convolution type inception block to extract the multi-scale feature information efficiently. Fig 3(c); shows our proposed block of multi-scale depthwise separable convolution. The proposed Xception block consists of different depthwise separable convolution kernel sizes, like 3 × 3, 5 × 5, and 7 × 7 followed by pointwise convolution with PReLU activation function, to reconstruct the SR image.

## 4 Experimental results

In this section, initially, we discuss the selection procedure of training and testing datasets with hyper-parameters. The training as well as testing datasets were downloaded from Kaggle website [80]. Afterwards, we have evaluated the quantitative as well as the qualitative performance in terms of PSNR/SSIM [81] and perceptual vision quality on five test datasets which are publicly available. Finally, we have compared the computational cost and processing speed of our proposed model in terms of PSNR versus the running time and network depth (number of *K* parameters).

### 4.1 Training datasets

The various sizes of the image datasets have been available for the training purposes to train the model for single image super-resolution. Yang et al. [23] and the Berkeley Segmentation Dataset (BSD300) [82] are commonly used image datasets, because these datasets are used by well-known SR methods, like VDSR [32], DRCN [33] and LapSRN [44] for the training purpose. In order to enhance the training dataset, data augmentation technique has been applied in terms of rotation and flipping. All the experimental evaluations were done on the original image and for data manipulation purposes, we used a programming language python 3.7.9, deep learning Keras 2.1.5 library supported back-end as Tensor Flow and PyTorch version 1.6.0. Various types of loss functions were also available to evaluate model performance. Deep learning-based CNN SR architecture has mostly used the mean square error (MSE) as the loss function. So, we have also used similar type of loss with our proposed method. Mathematically, the loss function may be calculated as:

$$\begin{array}{c} L\left(\theta \right)=\frac{1}{m}\sum_{i=1}^{m}F(\left(Y_{i};\theta \right)-X_{i}{)}^{2}, \end{array}$$ (7)

where *F*(*Y*_*i*_, *θ*) is the recovered output image, *X*_*i*_ is the high-quality HR images, *Y*_*i*_ is corresponding the low quality image, and the number of small size batches is the *m* in the training. In the training phase, we have used an adaptive momentum estimation optimizer (Adam) [83] having a 0.0004 initial learning rate with mini-batch size of 16. The process of training takes 200 epochs to converge the model properly. We train our model on a NVIDIA GeForce RTX2070 GPU, having 2.6 GHz Ci7-9750H CPU with 16 GB RAM under the Windows 10 operating system’s environment.

### 4.2 Testing datasets

We have assessed the performance of proposed network architecture on five standard datasets. The Set5 [84] dataset comprises of five images having different sizes like 228 × 228 and 512 × 512 pixels. The Set14 [85] images consist of different sizes of fourteen images. BSD100 [82] test dataset depends on 100 different natural scenes of images. Urban100 [86] is the challenging test image dataset having different frequency bands with detailed information. Manga109 [87] test image dataset depends on different comic type images with fine structures.

### 4.3 Implementation details

Under the Windows 10 operating system environment, our proposed approach was trained and tested with NVIDIA GeForce RTX2070 GPU with 16 GB RAM. We have trained our model on the scale enhancement factor of 2×, 4×, and 8× in Keras 2.1.5, PyTorch 1.6.0 and MATLAB 2018a framework.

### 4.4 Comparison with other state-of-the-art-methods

We compare the performance of our MXDSIR SR method with ground-truth HR image, including baseline method (Bicubic interpolation) and twelve other state-of-the-art methods are A+ [88], RFL [89], SelfExSR [86], SCN [41], SRCNN [40], FSRCNN [42], VDSR [32], DRCN [33], LapSRN [44], DRRN [47], MemNet [50], and MSISRD [65] by both objective PSNR/SSIM [81] and subjective measures. The summary of quantitative evaluation performed on five benchmark datasets as shown in Table 1. We can observe from Table 1, that our model achieves the best quantitative results in terms of PSNR/SSIM on enlargement factor 2× and 8×. The maximum and minimum range of the average PSNR improvement on scale factor 2× is 0.13dB to 4.12dB. Similarly, we also used another quality matrix to evaluate the performance of our proposed model is the SSIM. The minimum and maximum average range of SSIM improvement on scale factor 2× are in the range of 0.001 to 0.05. In the enlargement factor 4×, our model achieves the second-best performance as compared to other existing methods, though DRRN [47] and MSISRD [65] are the most comparable, but these models incur a higher computational complexity as they have more model parameters. Finally, our minimum and maximum improvement on challenging enlargement factor 8×, our range of the improvement in terms of average PSNR is 0.13dB to 1.73dB. Similarly, our model achieves minimum and maximum average SSIM improvement is 0.003 to 0.082.

**Table 1:** Presents benchmark results of the average value of PSNR/SSIM [81] for enlargement factor 2×, 4×, and 8× on Set5 [84], Set14 [85], BSD100 [82], Urban100 [86], and Manga109 [87] test datasets. Bold indicated results with red colors are the best values. The underlined results with blue color are $\underline{\text{second-best}}$ values.

Method	Scale	Para	Set5 PSNR/SSIM	Set14 PSNR/SSIM	BSD100 PSNR/SSIM	Urban100 PSNR/SSIM	Manga109 PSNR/SSIM	Average PSNR/SSIM
Bicubic	2×	-/-	33.69/0.931	30.25/0.870	29.57/0.844	26.89/0.841	30.86/0.936	30.52/0.884
A+ [88]	2×	-/-	36.60/0.955	32.32/0.906	31.24/0.887	29.25/0.895	35.37/0.968	32.96/0.922
RFL [89]	2×	-/-	36.59/0.954	32.29/0.905	31.18/0.885	29.14/0.891	35.12/ 0.966	32.86/0.920
SelfExSR [86]	2×	-/-	36.60/0.955	32.24/0.904	31.20/0.887	29.55/0.898	35.82/0.969	33.08/0.923
SCN [41]	2×	42	36.58/0.954	32.35/0.905	31.26/0.885	29.52/0.897	35.51/0.967	33.04/0.922
SRCNN [40]	2×	57	36.72/0.955	32.51/0.908	31.38/0.889	29.53/0.896	35.76/0.968	33.18/0.923
FSRCNN [42]	2×	12	37.05/0.956	32.66/0.909	31.53/0.892	29.88/0.902	36.67/0.971	33.56/0.926
VDSR [32]	2×	665	37.53/$\underline{0.959}$	33.05/0.913	31.90/$\underline{0.896}$	30.77/0.914	37.22/$\underline{0.975}$	34.09/0.931
DRCN [33]	2×	1775	37.63/$\underline{0.959}$	33.06/0.912	31.85/0.895	30.76/0.914	37.63/0.974	34.19/0.931
LapSRN [44]	2×	812	37.52/$\underline{0.959}$	33.08/0.913	31.80/ 0.895	30.41/0.910	37.27/0.974	34.02/0.930
DRRN [47]	2×	297	37.74/$\underline{0.959}$	33.23/$\underline{0.914}$	32.05/0.897	31.23/0.919	$\underline{37.92}$/0.976	34.43/$\underline{0.933}$
MemNet [50]	2×	677	37.78/$\underline{0.959}$	33.28/$\underline{0.914}$	32.08/0.897	$\underline{31.31}$/0.919	37.72/0.974	34.43/$\underline{0.933}$
MSISRD [65]	2×	240	$\underline{37.80}$/0.960	$\underline{33.84}$/0.920	$\underline{32.09}$/0.895	31.10/0.913	37.70/$\underline{0.975}$	$\underline{34.51/0.933}$
MXDSIR	2×	222	37.93/$\underline{0.959}$	33.87/0.920	32.12/0.897	31.33/0.918	37.93/0.976	34.64/0.934
Bicubic	4×	-/-	28.43/0.811	26.01/0.704	25.97/0.670	23.15/0.660	24.93/0.790	25.70/0.727
A+ [88]	4×	-/-	30.32/0.860	27.34/0.751	26.83/0.711	24.34/0.721	27.03/0.851	27.17/0.779
RFL [89]	4×	-/-	30.17/0.855	27.24/0.747	26.76/0.708	24.20/0.712	26.80/0.841	27.03/0.773
SelfExSR [86]	4×	-/-	30.34/0.862	27.41/0.753	26.84/0.713	24.83/0.740	27.83/0.866	27.45/0.787
SCN [41]	4×	42	30.41/0.863	27.39/0.751	26.88/0.711	24.52/0.726	27.39/0.857	27.32/0.782
SRCNN [40]	4×	57	30.50/0.863	27.52/0.753	26.91/0.712	24.53/0.725	27.66/0.859	27.42/0.782
FSRCNN [42]	4×	12	30.72/0.866	27.61/0.755	26.98/0.715	24.62/0.728	27.90/0.861	27.57/0.785
VDSR [32]	4×	665	31.35/0.883	28.02/0.768	27.29/0.726	25.18/0.754	28.83/0.887	28.13/0.804
DRCN [33]	4×	1775	31.54/0.884	28.03/0.768	27.24/0.725	25.14/0.752	28.98/0.887	28.19/0.803
LapSRN [44]	4×	812	31.54/0.885	28.19/0.772	27.32/$\underline{0.727}$	25.21/0.756	29.09/0.890	28.27/0.806
DRRN [47]	4×	297	31.68/$\underline{0.888}$	28.21/0.772	27.38/0.728	25.44/0.764	29.46/0.896	28.43/0.810
MemNet [50]	4×	677	$\underline{31.74}$/0.889	28.26/0.772	$\underline{27.40}$/0.728	$\underline{25.50}$/$\underline{0.763}$	29.42/0.894	28.46/$\underline{0.809}$
MSISRD [65]	4×	240	31.62/0.886	$\underline{28.51}$/$\underline{0.771}$	27.33/$\underline{0.727}$	25.42/0.757	31.61/0.891	28.90/0.806
MXDSIR	4×	222	32.37/$\underline{0.888}$	28.63/0.772	27.45/0.728	25.54/$\underline{0.763}$	$\underline{30.21}$/$\underline{0.895}$	$\underline{28.84/0.809}$
Bicubic	8×	-/-	24.40/0.658	23.10/ 0.566	23.67/0.548	20.74/0.516	21.47/0.650	22.68/0.588
A+ [88]	8×	-/-	25.53/0.693	23.89/0.595	24.21/0.569	21.37/0.546	22.39/0.681	23.48/0.617
RFL [89]	8×	-/-	25.38/0.679	23.79/0.587	24.13/0.563	21.27/0.536	22.28/0.669	23.37/0.607
SelfExSR [86]	8×	-/-	25.49/0.703	23.92/0.601	24.19/0.568	21.81/0.577	22.99/0.719	23.68/0.634
SCN [41]	8×	42	25.59/0.706	24.02/0.603	24.30/0.573	21.52/0.560	22.68/0.701	23.62/0.629
SRCNN [40]	8×	57	25.33/0.690	23.76/0.591	24.13/0.566	21.29/0.544	22.46/0.695	23.39/0.617
FSRCNN [42]	8×	12	25.60/0.697	24.00/0.599	24.31/0.572	21.45/0.550	22.72/0.692	23.62/0.622
VDSR [32]	8×	665	25.93/0.724	24.26/0.614	24.49/0.583	21.70/0.571	23.16/0.725	23.91/0.643
LapSRN [44]	8×	812	26.15/0.738	24.35/0.620	24.54/$\underline{0.586}$	21.81/$\underline{0.581}$	23.39/0.735	24.05/0.652
MemNet [50]	8×	677	26.16/0.741	$\underline{24.38}$/0.619	24.58/0.584	21.89/0.582	$\underline{23.56}$/$\underline{0.738}$	24.11/$\underline{0.653}$
DRCN [33]	8×	1775	25.93/0.723	24.25/0.614	24.49/0.582	21.71/0.571	23.20/0.724	23.92/0.643
MSISRD [65]	8×	240	$\underline{26.26}$/0.737	$\underline{24.38}$/$\underline{0.621}$	$\underline{24.73}$/$\underline{0.586}$	$\underline{22.53}$/0.582	23.50/0.738	$\underline{24.28/0.653}$
MXDSIR	8×	222	26.31/$\underline{0.740}$	24.42/0.622	24.77/0.587	22.91/0.582	23.63/0.739	24.41/0.654

**Fig 4:**
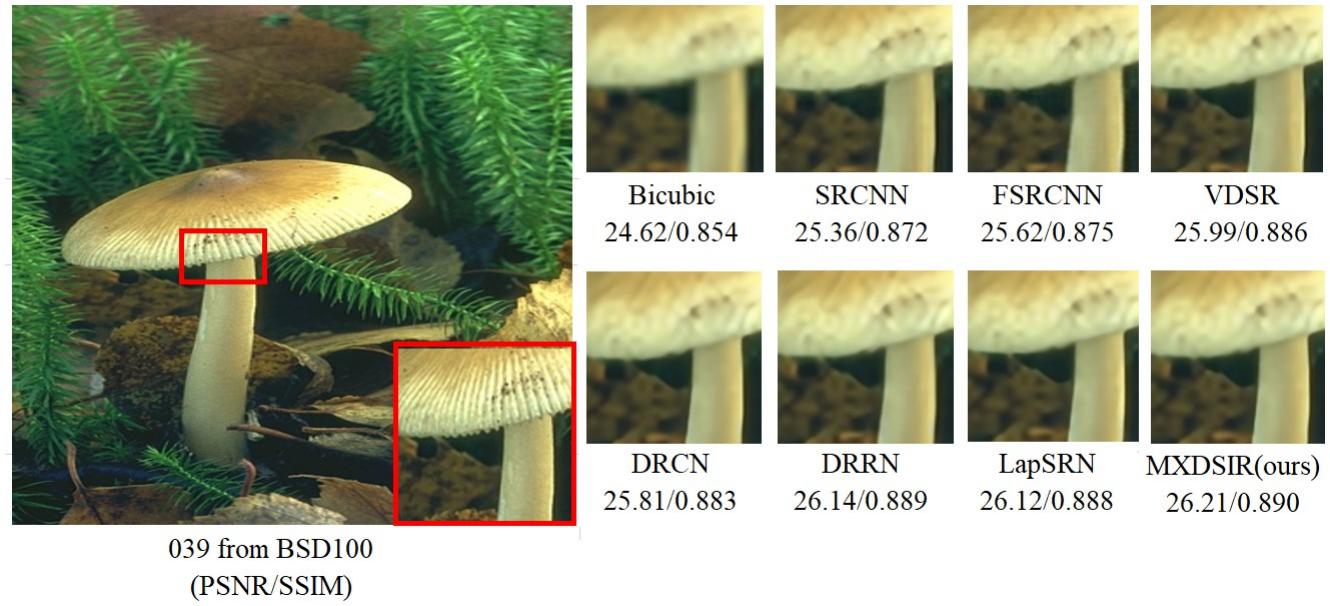
Visual performance of images with 4× enlargement factor of image 039 from BSD100 dataset.

**Fig 5:**
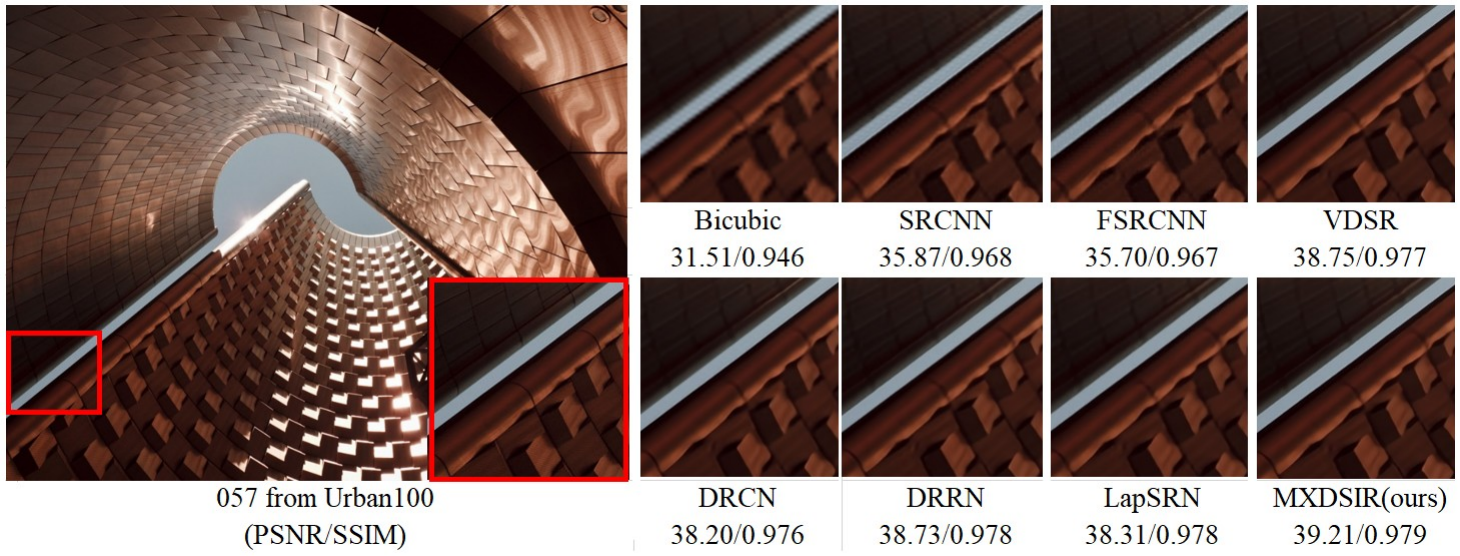
Visual performance of images with 4× enlargement factor of image 057 from Urban100 dataset.

**Fig 6:**
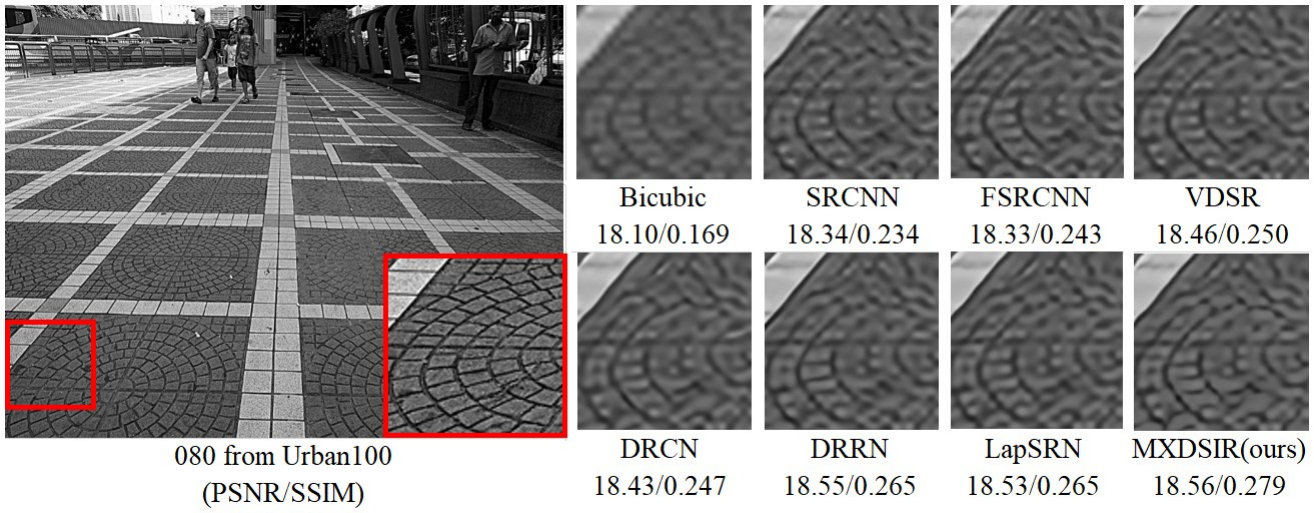
Visual performance of images with 8× enlargement factor of image 080 from Urban100 dataset.

**Fig 7:**
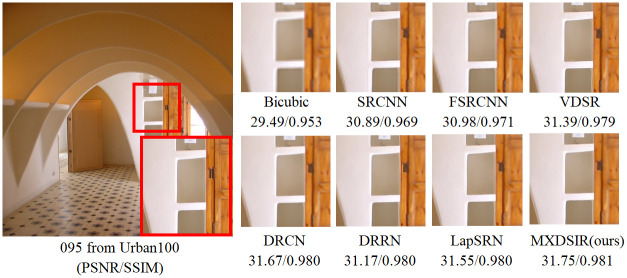
Visual performance of images with 8× enlargement factor of image 095 from Urban100 dataset.

**Fig 8:**
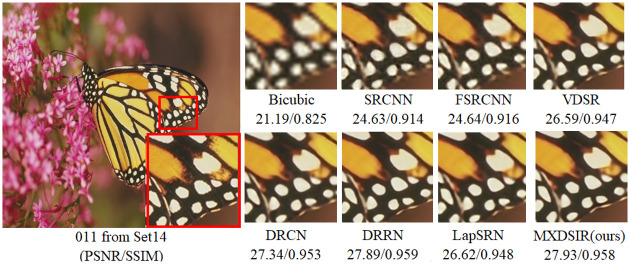
Visual performance of images with 8× enlargement factor of image 011 from Set14 dataset.

Apart from the quantitative comparison, the qualitative performance of our method and existing state-of-the-art methods are shown in Figs 4–8, were obtained from Huang [86] (https://github.com/jbhuang0604/SelfExSR) and [90] PLOS ONE Journal (https://doi.org/10.1371/journal.pone.0241313.g007).

From these images clearly observed that the baseline bicubic method cannot reconstruct any extra details information, but introduce the new noises in the image as well as more blurry results especially on enlargement scale factor 4× and 8×. The deep learning based image super-resolution approach, like SRCNN [40], FSRCNN [42] and VDSR [32] can produce, in some cases, fair reconstruction details from the original LR input image, but still results in blurry image contours due to their model designed in linear fashion (stacked layer side by side). In case of LapSRN [44] as well as family of deeper model, results are fair, but miss some edges and lines, because deeper model only relies on the single scale kernel. As we compare existing deeper model for image SR, our model achieves noticeable improvement in terms of perceptual quality, due to multiscale kernel used in the Xception block. The noticeable improvement observed in Fig 6; especially “080” image from Urban100 has excessive amount of artifacts, but our method produces sharper boundaries and richer textures with less amount of artifacts. Similar artifacts also observed on the image Figs 7 and 8 respectively.

In summary, our proposed method can achieve better quality improvement measured by PSNR, SSIM index, and visual image quality comparison compared to other methods. In the following sections, our proposed architecture provides a favorable trade-off in terms of computational cost and visual quality improvement.

### 4.5 Performance comparison in terms of the kernel size

**Fig 9:**
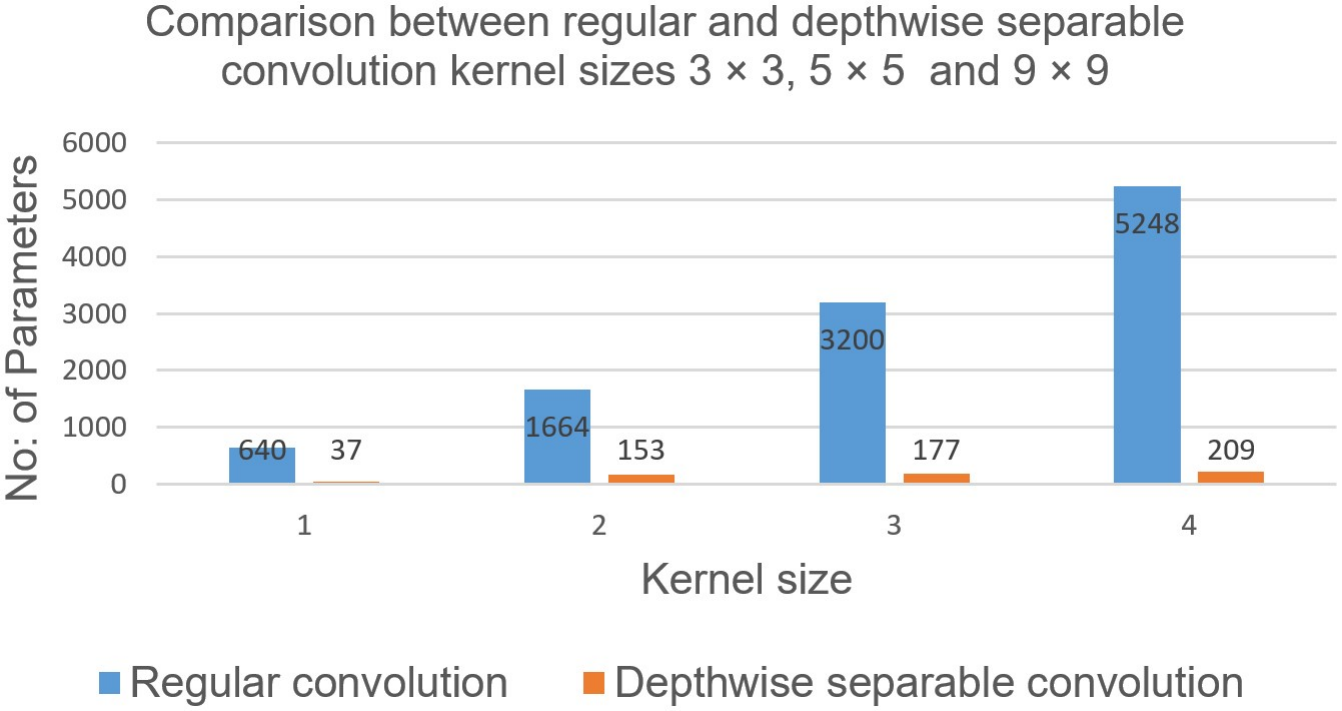
Complexity comparison between the regular convolution kernel versus the depthwise separable convolution kernel.

The size as well as the type of the convolution kernel plays a key role in terms of the model size and computational cost. In Fig 9; we have selected the two different convolution kernels, one is regular convolution kernel and the other is a depthwise separable convolution kernel, with the same 64 number of feature maps. Performance of our proposed depthwise separable convolution kernel is more computationally efficient as compared to the regular convolution kernel.

### 4.6 Comparison in terms of the number of the model parameters

**Fig 10:**
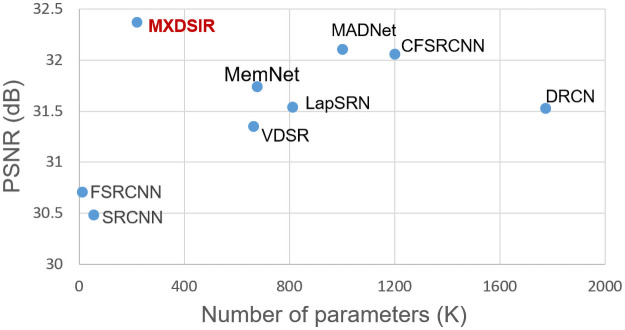
The performance comparison measurement on PSNR [81] versus the depth of the network (number of parameters). The performance results on the Set5 [84] dataset with scale factor 4×.

We have presented the complexity of the model related to network depth (number of parameters) versus PSNR [81] as shown in Fig 10. By using the depthwise separable convolution layer, our proposed model decreases the number of parameters as compared to other publicly available methods. Our MXDSIR method has parameters about 66% less than the VDSR [32], 87% less than the DRCN [33], 72% less than the LapSRN [44], 67% less than the MemNet [50], 74% less than MADNet [68] and 81% less than CFSRCNN [66].

### 4.7 Quantitative comparison in terms of run time versus PSNR

**Fig 11:**
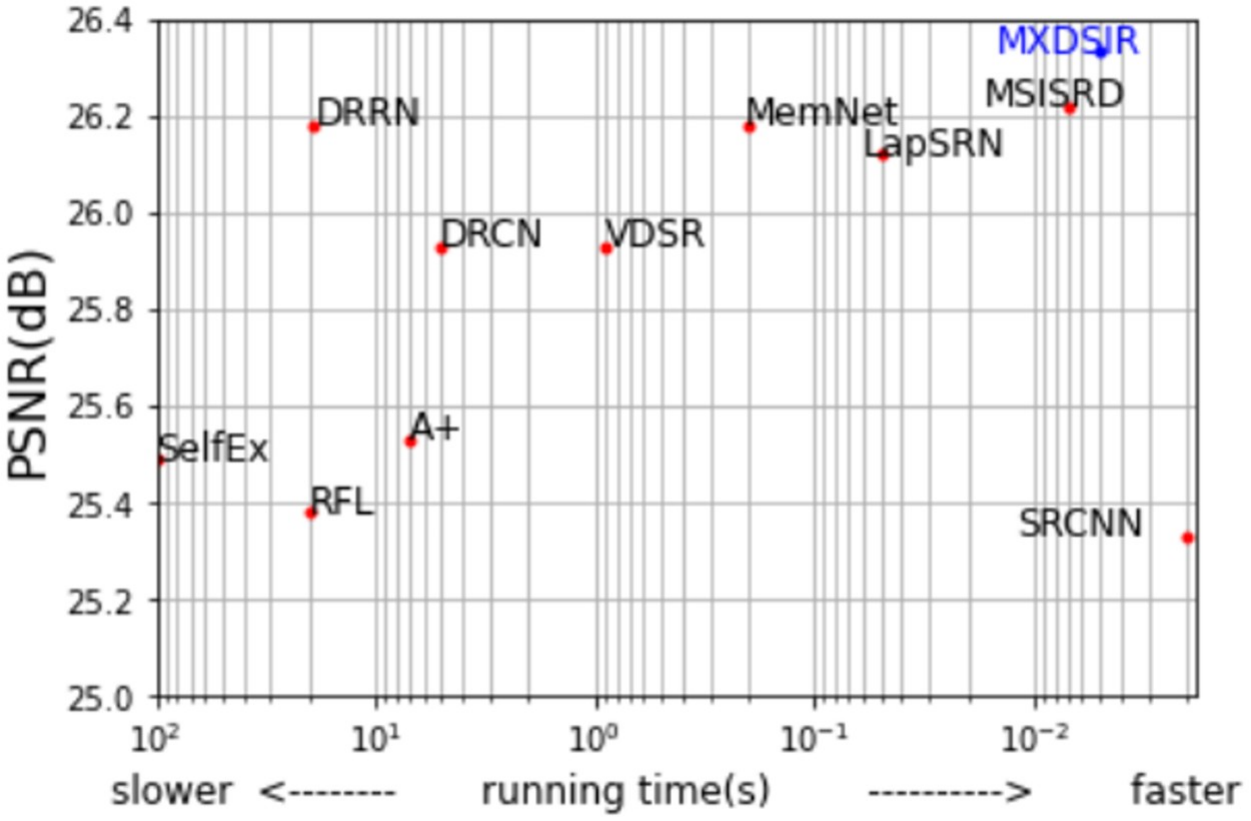
Quantitative comparison between the PSNR [81] performance vs. runtime on Set5 [84] scale 8× enlargement.

In this part, as shown in Fig 11; we have evaluated our method in terms of running or execution time versus PSNR [81]. As for the execution of time performance is concerned, we have used the public access codes given by the authors to evaluate the state-of-the-art methods with 2.6 GHz Ci7-9750H CPU 16GB RAM. The comparative analysis between the execution of time and performance on the Set5 [84] dataset for 8× SR reveals that our method is 0.16 dB higher than LapSRN [44] on PSNR [81] and, approximately, 10 times faster than LapSRN [44].

## 5 Conclusion

In this paper, we have presented fast and computationally efficient Xception based residual CNN network architecture for image SR to extract the features information locally as well as globally from the input LR image, and to generate the HR output image. The proposed network architecture used the two ResNet blocks and three Xception block, which is adopted from the ResNet and GoogLeNet to recover several features during the extraction and reconstruction stages. The proposed technique ensured that the network shows fast convergence speed and low computational cost, by replacing the interpolation technique with the learned transposed convolution layer and regular convolution operation with the depthwise separable convolution. Furthermore, our network architecture is relatively simple and well designed for images and computer vision tasks. Extensive experimental results on different image datasets not only provides satisfactory results on the performance of image SR quantitatively but also have favorable results in terms of complexity and provided visual pleasing quality as compare to the existing state-of-the-art SR methods.
